# Biological scoring system for early prediction of acute bowel ischemia after cardiac surgery: the PALM score

**DOI:** 10.1186/s13613-018-0395-5

**Published:** 2018-04-18

**Authors:** Elie Zogheib, Cyril Cosse, Charles Sabbagh, Simon Marx, Thierry Caus, Marc Henry, Joseph Nader, Mathurin Fumery, Michael Bernasinski, Patricia Besserve, Faouzi Trojette, Cedric Renard, Pierre Duhaut, Said Kamel, Jean-Marc Regimbeau, Hervé Dupont

**Affiliations:** 1 Cardio-thoracic and Vascular Intensive Care Department, Amiens University Hospital, Amiens, France; 2 INSERM U1088, Jules Verne University of Picardie, Amiens, France; 3 Department of Digestive and Oncological Surgery, Amiens University Hospital, Amiens, France; 4 SSPC (Simplification des Soins Patients Chirurgicaux Complexes) BQR Unit of Clinical Research, Jules Verne University of Picardie, Amiens, France; 5 Department of Cardiac Surgery, Amiens University Hospital, Amiens, France; 6 Department of Gastroenterology, Amiens University Hospital, Amiens, France; 7 Department of Radiology, Amiens University Hospital, Amiens, France; 8 Department of Internal Medicine and RECIF, Amiens University Hospital, Amiens, France; 9 Department of Biochemistry, Amiens University Hospital, Amiens, France

**Keywords:** Scoring system, Bowel ischemia, Cardiac surgery, Procalcitonin

## Abstract

**Background:**

Bowel ischemia is a life-threatening emergency defined as an inadequate vascular perfusion leading to bowel inflammation resulting from impaired colonic/small bowel blood supply. Main issue for physicians regarding bowel ischemia diagnosis lies in the absence of informative and specific clinical or biological signs leading to delayed management, resulting in a poorer prognosis, especially after cardiac surgery. The aim of the present series was to propose a simple scoring system based on biological data for the diagnosis of bowel ischemia.

**Methods:**

In a retrospective monocentric study, patients admitted in cardiac ICU, after cardiovascular surgery, were screened for inclusion. According to a 1:2 ratio (case–control), matching between two groups was based on sex, type of cardiovascular surgery, and the operative period (per month). Patients were divided into two groups: “ischemic group” which corresponds to patients with confirmed bowel ischemia and “non-ischemic group” which corresponds to patients without bowel ischemia. Primary objective was the conception of a scoring system for the diagnosis of bowel ischemia. Secondary objectives were to detail the postoperative morbidity and the diagnostic features for the distinction between acute mesenteric ischemia and ischemic colitis.

**Results:**

Forty-eight patients (1.3%) had confirmed bowel ischemia (“ischemic group”). According to the 2:1 matching, 96 patients were included in the “non-ischemic group.” Aspartate aminotransferase > 449 UI/L, lactate > 4 mmol/L, procalcitonin > 4.7 μg/L, and myoglobin > 1882 μg/L were found to be independently associated with bowel ischemia. Based on their respective odds ratios, points were assigned to each item ranging from 4 to 8. AUROCC [95% confidence interval] of the scoring system to diagnose bowel ischemia was 0.93 [0.91–0.95], *p* < 0.001. The optimal threshold after bootstrapping was ≥ 14 points; this yielded a sensitivity of 85.4%, a specificity of 94.8%, a positive likelihood ratio of 16.42, a negative likelihood ratio of 0.15, a Youden’s index of 0.802, and a diagnostic odds ratio of 106.62.

**Conclusions:**

A biological scoring system based on PCT, ASAT, lactate, and myoglobin measurement allows the diagnosis of bowel ischemia after cardiac surgery with high accuracy. This score could help clinician to propose an early diagnosis and an early treatment in this high mortality disease.

**Electronic supplementary material:**

The online version of this article (10.1186/s13613-018-0395-5) contains supplementary material, which is available to authorized users.

## Background

Gastrointestinal complications after cardiovascular surgery are rare conditions occurring in 0.3–3% of cases but are associated with a major impact on patient’s management, especially in terms of morbidity and mortality [1]. Among these conditions, the most frequent one is bowel ischemia (including mesenteric ischemia and ischemic colitis).

Bowel ischemia is a life-threatening emergency defined as an inadequate vascular perfusion leading to bowel inflammation resulting from impaired colonic/small bowel blood supply. The suspected incidence for bowel ischemia is one hospitalization out of 1000 in the general population and complicated 1% of cardiac surgery [2, 3]. The incidence of ischemic colitis ranges from 4.5 to 44 cases per 100,000 persons per year, and it complicated 1% of the cardiac surgery [4]. The associated mortality reported in the literature is more than 75% for mesenteric ischemia [5] and depends on the mucosal ischemic damage stage of the colon (Favier’s classification): Stage I is an ischemia limited to the mucosa (0% mortality), stage II is an ischemia extended to the muscularis mucosa with large ulcerations, and stage III is a transmural ischemia with necrosis of the muscularis and possible perforation (mortality > 75%) [6]. From a pathophysiological point of view, bowel ischemia is associated with mucosal ulceration, inflammation, and hemorrhage, with a positive feedback due to reperfusion in case of blood flow restoration [7–9].

The main issue for physicians about bowel ischemia diagnosis lies in the absence of informative and specific clinical or biological signs leading to delayed management and resulting in a poorer prognosis, especially after cardiac surgery. Indeed, initial symptoms such as pain or abdominal distention are hard to detect in this intensive care unit (ICU) patient population. To overcome this issue, several teams have looked for risk factors for bowel ischemia after cardiac surgery, including catecholamine use, blood transfusion, emergency cardiac surgery, and acute renal failure [10]. Nevertheless, despite the presence of these risk factors, bowel ischemia remains a diagnostic challenge for physicians.

The aim of the present study was to propose a simple scoring system based on biological data for diagnosis of bowel ischemia.

## Patients and methods

### Population and study design

From July 1, 2006, to December 31, 2013, patients hospitalized in the Cardio-thoracic and Vascular Intensive Care Departments (University Hospital) after cardiovascular surgery were screened for inclusion from a local database in this single-center, retrospective, case–control study. Patients were divided into two groups: the case group (“ischemic group”) which corresponds to patients with confirmed bowel ischemia (CT scan, endoscopy and/or laparotomy). The patients of the non-ischemic group were randomly extracted from the local database. Matching between the two groups was based on sex, type of cardiovascular surgery, and the operative period (per month) according to a 1:2 ratio (case–control).

This was a retrospective, non-interventional, observational study reporting data of a large cohort and not about an individual patient. As such, according to French legislation, neither informed consent nor approval of the ethics committee was required to use data from patient records. All data were collected anonymously from medical records only. Data representing patient identifiers were not collected.

### Objectives of the study

#### Primary objective

The study’s primary objective was the conception of a scoring system for the diagnosis of bowel ischemia.

#### Secondary objectives

The secondary objectives were to detail the postoperative morbidity and the diagnostic features for the distinction between acute mesenteric ischemia and ischemic colitis.

### Inclusion/exclusion criteria in the ischemic group

The inclusion criteria were as follows: (1) age 18 or over; (2) history of cardiovascular surgery; and (3) histologically confirmed diagnosis of bowel ischemia (in the ischemic group).

### Data collected

The following items were retrospectively retrieved from the patients’ medical records and transferred into a Microsoft Excel^®^ file: demographic information, surgical data (aortic, mitral or tricuspid cusp replacement or repair, coronary artery bypass, clamping duration, bypass duration, or catecholamine or vasoactive drug use), laboratory blood sample results, comorbidities (diabetes, hypertension, cardiopathy, neurological disorders, chronic obstructive pulmonary disease and peripheral vascular disease), vital status (the American Society of Anaesthesiology or ASA score), and the postoperative course (vasopressors need, RRT, fluid balance, etc.).

### Patient management

Following cardiac surgery, all patients were admitted to the cardiac intensive care unit and the severity of their disease was assessed using the Simplified Acute Physiology Score or SAPSII score. In the postoperative period, standardized systematic blood samples were collected at H0, H3, H6, and postoperative days 1 and 2 for ionogram analysis and protidemia and creatinine assessment; a blood panel, liver function tests (ASAT, alanine aminotransferase (ALT), bilirubin), an arterial blood gas analysis including measurement of lactate and venous blood gas, C reactive protein (CRP), PCT, and cardiac panel (myoglobin, troponin, creatine kinase) were also performed. Some additional blood samples may have been collected depending on the patient’s clinical status.

Bowel ischemia was suspected clinically (abdominal pain and/or distention, rectal bleeding, oliguria or anuria, persistent hypotension despite fluid loading and increasing vasopressor requirements), and/or biologically (metabolic acidosis with increased lactate serum level at least more than 3.0 mmol/l). In these patients with high suspicion of bowel ischemia, an abdominal CT scan (assessed by an experienced radiologist) was performed [11]. When mesenteric ischemia was suspected, exploratory laparotomy was carried out. When the CT scan was normal, or if ischemic colitis was suspected, a colonoscopy was done. The choice of one examination over another resulted from a multidisciplinary decision.

### Statistical analysis and ethical considerations

Quantitative variables including biological values were expressed as median (range) or mean ± standard deviation (SD) in case of Gaussian distribution. Qualitative variables (especially for patient’s demographic) were expressed in terms of counts (percentage). Univariate analyses were based on the t test for paired data (for quantitative variables) and the Mac Nemar Chi-squared test (for qualitative variables). Diagnostic factors were expressed as odds ratio (OR) (95% confidence interval) (95% CI) and *p* value. The patients of the non-ischemia group were randomly extracted from the local database. Matching between the two groups was based on sex, type of cardiovascular surgery and the operative period (per month) according to a 1:2 ratio (case–control).

To conceive the scoring system, we generated a receiver operating characteristic (ROC) curve for each biological value and calculated the area under the ROC curve (AUROCC) with its 95% confidence interval. From a multivariate analysis with a stepwise logistic regression according to the Wald method, independent diagnostic factors were identified. These factors were weighed according to their odds ratio for the conception of the score.

Predictive power was characterized by sensitivity (Se), specificity (Sp), threshold, positive and negative likelihood ratios (LR+ and LR−), Youden’s index (*I*, calculated as *I* = Se + Sp − 1), and diagnostic odds ratio (DOR, calculated as follows: DOR = (Se*Sp)/(1 − Se)*(1 − Sp)). To ensure the robustness of our data, the calculations were based on a 1000-patient population bootstrapped (sampling with replacement) from the original population. To obtain the bootstrapped population (1000 patients), seven samplings with replacement were performed from the original dataset (*n* = 144 patients). For each sampling, the number of replications per patient was random, varying from 1 replicate to 4 replicates. The aim of the bootstrap was to increase the sample size representative of the original population, with a robust method and a low impact of outliners.

Statistical analysis was performed with SAS software (version 9.2; SAS Institute Inc., Cary, NC, USA) and PASW software (version 22, SPSS INC., Chicago, IL, USA). *p* value ≤ 0.05 was considered as statistically significant. The study’s results were reported in accordance with the “STARD” statement.

## Results

### Population

**Fig. 1:**
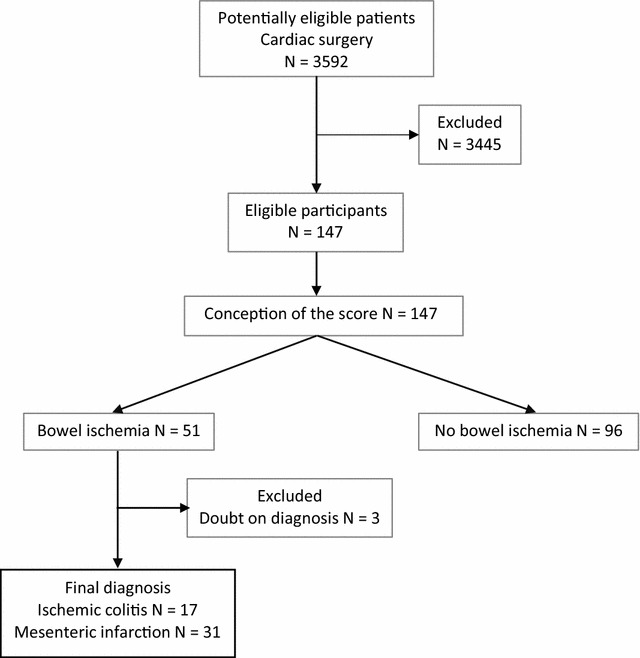
Study flowchart (according to the STARD guidelines) describing the whole eligible population in the postoperative of cardiac surgery patients during the period of the study, leading to the final population studied in both groups

During the study period, 3.592 patients underwent elective cardiac surgery with or without cardiopulmonary bypass. Among these patients, 48 patients (1.3%) had confirmed bowel ischemia and constituted the ischemic group. In this ischemic group, there were 31 mesenteric ischemia and 17 ischemic colitis. According to the 2:1 matching, 96 patients were included in the non-ischemic group (Fig. 1). The population was well balanced in terms of demographic data and pre- and peroperative data except for emergency surgery, use of vasopressor, volume of macromolecules ≥ 500 mL, and transfusion, which were more frequent in the ischemic group. Conversely, the volume for fluid loading was lower in the ischemic group (Table 1; Additional file 1: Table S1).

**Table 1:** Patients preoperative and peroperative characteristics

Variables	Ischemic group (*n* = 48)	Non-ischemic (*n* = 96)	*p* value
*Preoperative characteristics*			
Age, years, mean ± SD	73.6 ± 9.2	73.2 ± 9.2	0.80
Male, *n* (%)	27 (56.3)	54 (56.3)	0.99
Body mass index, kg/m^2^, mean ± SD	27.7 ± 4.9	27.2 ± 4.4	0.51
Hypertension, *n* (%)	40 (83.3)	66 (62.3)	0.06
Coronaropathy, *n* (%)	26 (54.2)	57 (59.4)	0.55
Dyslipidemia, *n* (%)	26 (54.2)	46 (47.9)	0.48
Atrial fibrillation, *n* (%)	15 (31.3)	24 (25.0)	0.35
Smoking, *n* (%)	17 (54.8)	30 (31.3)	0.66
Peripheral arterial disease, *n* (%)	6 (12.5)	10 (10.4)	0.71
Diabetes, *n* (%)	7 (14.6)	28 (29.4)	0.05
ASA score 3–4, *n* (%)	48 (100)	94 (97.9)	0.56
Emergency surgery, *n* (%)	14 (29.2)	13 (13.5)	*0.02*
*Peroperative characteristics*			
Extracorporeal circulation (ECC), *n* (%)	39 (81.3)	82 (85.4)	0.52
ECC duration, min, mean ± SD	112.4 ± 63.0	100.1 ± 62.2	0.27
Aortic clamping duration, min ± SD	59.8 ± 47.0	66.4 ± 43.0	0.40
Spontaneous fibrillation, *n* (%)	33 (68.8)	65 (67.7)	0.90
Electrical cardioversion, *n* (%)	15 (31.2)	31 (32.3)	0.90
Loading volume, ml, mean ± SD	1915.9 ± 696.5	2573.9 ± 823.0	*0.005*
Use of macromolecules, *n* (%)	27 (56.3)	54 (56.3)	0.99
Volume of macromolecules ≥ 500 mL, *n* (%)	16 (33.3)	15 (15.6)	*0.015*
Use of ephedrin, *n* (%)	11 (22.9)	20 (20.8)	0.08
Use of neosynephrin, *n* (%)	11 (22.9)	14 (14.6)	0.21
Use of catecholamines, *n* (%)	27 (56.3)	21 (21.4)	*0.005*
Use of a cell saver, *n* (%)	46 (95.8)	93 (96.9)	0.75
Transfusion, *n* (%)	21 (43.8)	25 (26.0)	*0.03*

### Conception of a scoring system for the diagnosis of bowel ischemia

#### Identification of the parameters

**Fig. 2:**
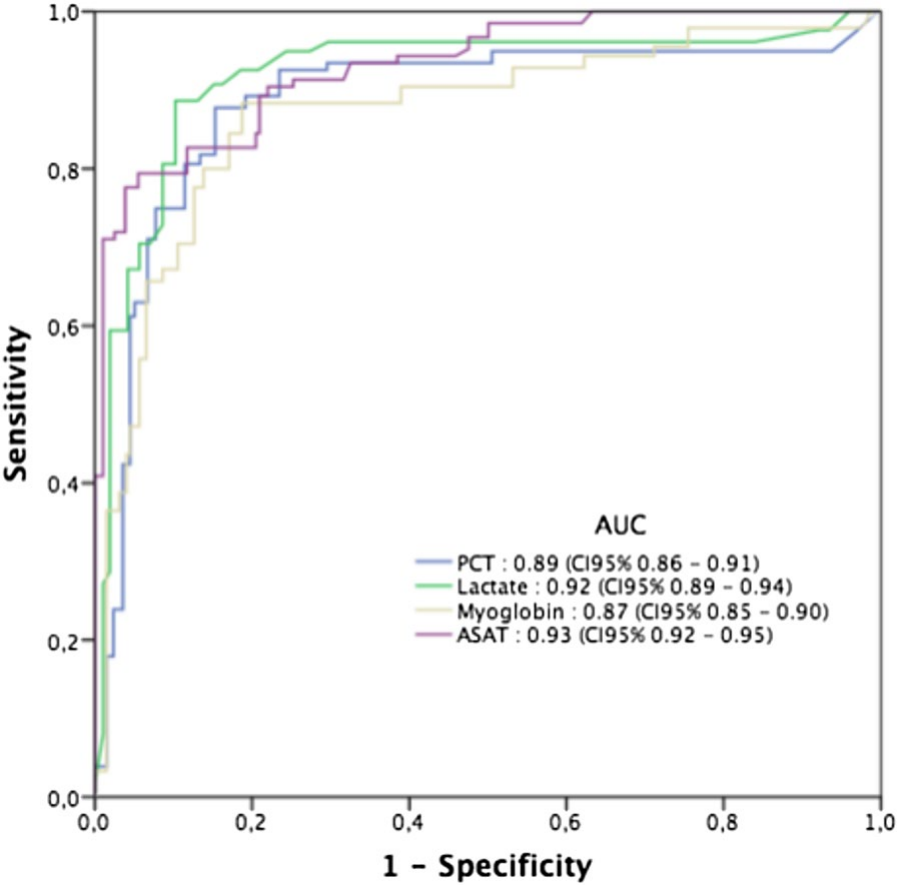
Comparison of the ROC curves showing the sensitivity and specificity of each marker (PCT, ASAT, lactate and myoglobin) for predicting the diagnosis of bowel ischemia

Considering univariate analysis, the parameters relevant for the diagnosis of acute mesenteric ischemia are presented in Table 2 and Fig. 2. Patients in the ischemic group presented a higher lactate level and a lower creatinine clearance. Based on diagnosis suspicion, all collected parameters were increased, in particular lactate, PCT, and CRP.

**Table 2:** Biological features for the diagnosis of acute bowel ischemia

Variables	Ischemia group (*n* = 48)	Non-ischemia (*n* = 96)	*p* value
*After cardiovascular surgery*			
ASAT, IU/L, median (range)	106 (16–1980)	80 (14–1372)	0.30
ALAT, IU/L, median (range)	41 (3–1022)	35 (4–866)	0.69
Lactate, mmol/L, mean ± SD	2.8 ± 2.5	1.6 ± 0.9	*0.005*
Creatinine clearance, mL/min, mean ± SD	62.3 ± 25.8	80.4 ± 27.3	*0.005*
*Based on diagnosis suspicion*			
Leukocytes, ×1000/mm^3^, mean ± SD	15.4 ± 7.6	11.7 ± 9.2	*0.02*
ASAT, IU/L, median (range)	2610 (27–12,964)	120 (13–4321)	*< 0.001*
ALAT, IU/L, median (range)	1200 (13–4464)	67 (9–2227)	*< 0.001*
Lipase, IU/L, median (range)	74 (4–956)	29 (8–235)	*0.006*
Amylase, IU/L, median (range)	553 (18–5981)	159 (9–1723)	*< 0.001*
Potassium, mmol/L, mean ± SD	5.8 ± 1.0	4.6 ± 0.7	*0.005*
Troponin, μg/L, median (range)	20 (0.02–361)	6 (0.01–95.29)	*0.01*
Myoglobin, μg/L, median (range)	8690 (107–94,187)	953 (50–25,480)	*0.001*
Creatine kinase, μg/L, median (range)	2402 (16–15,442)	1073 (12–19,197)	*0.005*
Lactate, mmol/L, mean ± SD	7.7 ± 5.7	3.1 ± 2.8	*0.005*
Procalcitonin (PCT), μg/L, median (range)	16 (0.45–103.93)	2.5 (0.07–57.15)	*0.001*
C reactive protein, mg/L, mean ± SD	194.3 ± 104.1	130.8 ± 81.3	*0.005*

The scoring system is presented in Table 3. Among all clinical and biological variables tested, ASAT (> 449 UI/L), lactate (> 4 mmol/L), PCT (> 4.7 μg/L) and myoglobin (> 1882 μg/L) were found to be independently associated with bowel ischemia. Thus, only four parameters were found as possible independent factors of acute mesenteric ischemia in the multivariate logistic regression model OR (95% CI): PCT 8.5 (1.9–27.9), ASAT 7.7 (1.9–35.2), lactate 7.1 (1.7–28.3), and myoglobin 3.5 (1.0–16.6). Based on their odds ratios, points were assigned to each item ranging from 4 to 8 points, using the best sensitivity and specificity according to the better likelihood ratio (Table 4).

**Table 3:** Scoring system for the diagnosis of acute bowel ischemia

Biological parameters	Score (points)	OR	CI 95%	*p* value	Threshold	CI 95%
PCT (μg/L)	8	8.5	1.9–27.9	0.004	4.7	2.8–10.8
ASAT (IU/L)	8	7.7	1.9–35.2	0.005	449	146–628
Lactate (mmol/L)	7	7.1	1.7–28.3	0.008	4	3.8–5.1
Myoglobin (μg/L)	4	3.5	1.0–16.6	0.048	1882	1838–5131

**Table 4:** Best sensitivity and specificity according to the better likelihood ratio, leading to the points of the score for the diagnosis of acute bowel ischemia

Variable	Se	95% CI	Sp	95% CI	LR+	95% CI	LR−	95% CI
PCT (μg/L)	85.42	72.2–93.9	84.37	75.5–91.0	5.47	3.4–8.8	0.17	0.09–0.3
ASAT (IU/L)	75	60.4–86.4	95.83	89.7–98.9	18	6.8–47.6	0.26	0.2–0.4
Lactate (mmol/L)	87.5	74.8–95.3	90.62	82.9–95.6	9.31	5.0–17.5	0.14	0.07–0.3
Myoglobin (μg/L)	87.5	74.8–95.3	81.25	72.0–88.5	4.67	3.0–7.2	0.15	0.07–0.3

*Se* sensitivity, *Sp* specificity, *LR*+ positive likelihood ratio, *LR*− negative likelihood ratio

#### Predictive characteristics of the scoring system

**Fig. 3:**
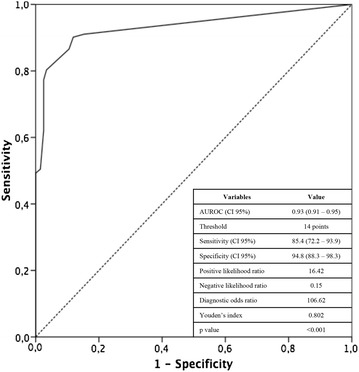
ROC curve showing the sensitivity and specificity of the PALM score including the four markers (PCT, ASAT, lactate, and myoglobin) for predicting the diagnosis of bowel ischemia. The AUROCC [95% confidence interval] was 0.93 [0.91–0.95]; *p* < 0.001

The predictive characteristics of the scoring system are presented in Table 3 and the associated ROC curve in Fig. 3.

The AUROCC [95% confidence interval] was 0.93 [0.91–0.95]; *p* < 0.001. The optimal threshold for predicting acute mesenteric ischemia after bootstrapping was ≥ 14 points; this yielded a Se of 85.4%, a Sp of 94.8%, a LR+ of 16.42, a LR− of 0.15, an I of 0.802 and a DOR of 106.62 (Table 4).

### Postoperative morbidity and mortality

In the ischemia group, the postoperative mortality rate was 81.3% (*n* = 39) vs. 7.3% (*n* = 7) in the non-ischemia group. Patients in the ischemia group presented more frequent multiple organ failure (MOF) including pneumonia (35 vs. 20.8%), acute kidney failure (89.6 vs. 50%) requiring renal replacement therapy (50 vs. 6.3%), leading to a tenfold greater mortality (81.3 vs. 7.3%, *p* < 0.001), then in the non-ischemia group, respectively (Additional file 2: Table S2).

### Diagnostic features for the distinction between acute mesenteric ischemia and ischemic colitis

The diagnostic features are presented in Additional file 3: Table S3. Only rectal bleeding and colonoscopy were more frequently found in patients with ischemic colitis. A surgical exploration was more often performed in case of suspicion of acute mesenteric ischemia (93.5% of cases). Other clinical, biological, and paraclinical features were well balanced between the two populations.

## Discussion

To our knowledge, this is the first study to conduct a matched-pair analysis to propose a simple biological scoring system for the diagnosis of bowel ischemia after cardiac surgery. This simple score included daily practice biological parameters (PCT, ASAT, lactate, and myoglobin). With a score of 14 points, the predictive characteristics for bowel ischemia included high a sensitivity of 85.4%, a specificity of 94.8%, high LR+ (> 4) and low LR− (< 0.5). This is of high interest since up today, the diagnosis of bowel ischemia is based on very unspecific clinical or radiological signs that can lead to endoscopy or surgery to confirm or not the diagnosis and is still associated with a high mortality rate. In our study, the incidence of bowel ischemia was 1.33% with an overall mortality of 81%. Previous studies reported an incidence of 0.3–3% and described mortality rates between 30 and 100% in patients with mesenteric ischemia after cardio pulmonary bypass [12, 13]. The use of a simple scoring system to identify bowel ischemia is clinically interesting; consideration of these combined four parameters might enable to accurately help in the identification of population at risk of bowel ischemia earlier and thus lead to early investigations (CT scan and colonoscopy) and guide patient management before the occurrence of non-reversible lesions and organ failure (patient purpose).

This study blends in the contemporary consideration about the place of biomarkers in daily practice and especially in the diagnosis of digestive emergencies. ASAT and myoglobin were identified as diagnostic parameters in this study. It is quite puzzling as the available literature in animal and human trials is torn between interest and futility for the diagnosis of bowel ischemia [14–17]. Among the most investigated biomarkers in the field of bowel ischemia, two potential candidates are already available for physicians (PCT and lactate) and are the subjects of discussions as reported in the existing literature. Firstly, PCT has recently been shown as a prognostic and “therapeutic” guide for the management of ischemic bowel disease such as ischemic colitis and adhesion related small bowel obstruction [8, 18]. In this sense, the present results are concordant with previous findings emphasizing the potential value of serum PCT as a systemic biomarker of intestinal ischemic damage. Moreover, its diagnostic performance for bowel ischemic disorders has already been reported by Nagata et al. [19], who showed that PCT helps rule out ischemic disorders after cardiac surgery. In pathophysiological terms, the PCT secretion induced by the pro-inflammatory mediators IL6 and TNFα is enhanced in ischemic diseases, as a result of malperfusion of the intestine with subsequent loss of the epithelial barrier function and bacterial translocation and endotoxin release, important phenomenon in the colon [20–22]. The use of PCT as a single marker as, nevertheless some limitations as reported by Cosse et al. [8], PCT levels may be elevated by some non-ischemic phenomena (cardiac arrest, drug reaction with eosinophilia and systemic symptoms syndrome, heat wave, etc.) and decreased by others phenomena (previous effective antibiotic therapy, tuberculosis, etc.).

Secondly, lactate resulting from the anaerobic glycolysis occurring in case of decreased vascular perfusion and oxygenation, i.e., in case of ischemia, has also been described as a predictor of the outcomes of bowel ischemia and so may contribute to the diagnosis of acute mesenteric ischemia (AMI). However, in the literature, its performance appears disappointing, because normal lactate concentrations cannot exclude the diagnosis as suggested by several teams [17, 23]. This trend was supported by a systematic review of the serological markers for human intestinal ischemia reporting low sensitivity and specificity [24] to the point that they are not recommended by the European Society for Trauma and Emergency Surgery (ESTES) guidelines for the diagnosis of intestinal ischemia [25].

The clinical interest of this score for the diagnosis of bowel ischemia may be attributed to its impact on the management of patients, especially those with a high risk of bowel ischemia after cardiovascular surgery; earlier CT scan and colonoscopies with a concomitant decrease in mortality due to a more aggressive management [26].

The use of this score associate with the use of a systematic colonoscopy, especially when the PALM score is > 14 is a potential forward step to a better management of patient with a high risk of bowel ischemia (as after cardiac surgery). There is also a necessity to homogenize the management of these patients to decrease the risk of death. Recently, Moszkowicz et al. [27] proposed an algorithm of management of the ischemic colitis based on a colonoscopic classification (Favier’s classification). Surgical resection is proposed to all patients with a stage III ischemic colitis and to stage II patient with multiorgan failure. By using this classification, the authors report 51% of mortality. These outcomes could be improving by a quicker diagnosis of the ischemic colitis and the PALM has a potential indication here. Nuzzo et al. have published an algorithm of management of the mesenteric ischemia which is a lifesaving strategy that required a quick management based mainly on the abdominal CT scan. In this strategy, the PALM score could also have an interest [28, 29].

Our study has several limitations. Firstly, only 147 patients were included, 48 of whom because of bowel ischemia. Secondly, several etiologies of ischemia were retained (mesenteric ischemia, ischemic colitis) with different physiopathology and prognosis. Because one of the diagnostic problems of intestinal ischemia is the diversity in pathophysiological processes, we aimed to improve diagnosis of intestinal ischemia no matter the localization. The occlusive or non-occlusive aspect of ischemia will not be directed by this score, neither mesenteric nor colonic ischemia. However, at our knowledge, PALM score had a high accuracy between the biomarkers and intestinal infarction, that failed to demonstrate recently other specific biomarkers as D-lactate, intestinal fatty-acid-binding protein (i-FABP), and smooth muscle actin (SMA) [30]. According to the matching condition, we recognize the presence of more emergency surgery, more transfusion, and less fluid administration in the ischemia group, what could be related the etiologies of the low blood flow leading to ischemia digestive without modifying neither the biological markers of this ischemia nor the accuracy value of our biological score. Thirdly, from a methodological point of view, this study was designed to look for a high true negative rate. If the chosen approach would have been based on the highest sensitivity, the threshold of each parameter would have been decreased with a resulting sensitivity and specificity of the PALM score of respectively 90 and 80% instead of 85 and 95%.

Despite these shortcomings, the present study constitutes one of the largest available analyses of a patient population with bowel disorders and enables us to draw up hypotheses for future studies especially within the framework of a prospective evaluation as part of a series of exams to help in decision making.

## Conclusion

A biological scoring system based on PCT, ASAT, lactate, and myoglobin allows the diagnosis of bowel ischemia after cardiac surgery with high accuracy. This score could help clinician to propose an early diagnosis and to propose an early treatment in this high mortality disease.

## Additional files

**Additional file 1: Table S1.** Type of cardiac surgery.

**Additional file 2: Table S2.** Postoperative course.

**Additional file 3: Table S3.** Features for the distinction between mesenteric ischemia and ischemic colitis.

## Abbreviations

AF: atrial fibrillation
ALAT: alanine aminotransferase
AMI: acute mesenteric ischemia
ASA: american society of anesthesiologists
ASAT: aspartate aminotransferase
AUROC: area under the receiver operating characteristic
AUROCC: area under the receiver operating characteristic curve
BMI: body mass index
CI: confidence interval
CRP: C reactive protein
DOR: diagnostic odds ratio
ESTES: European Society for Trauma and Emergency Surgery
HTA: hypertension
ICU: intensive care unit
LR: likelihood ratio
MOF: multiorgan failure
OR: odds ratio
PCT: procalcitonin
ROC: receiver operating characteristic
RRT: renal replacement therapy
SAPS II: Simplified Acute Physiology Score
SD: standard deviation
Se: sensitivity
Sp: specificity
