# Supplementary material for: Biological scoring system for early prediction of acute bowel ischemia after cardiac surgery: the PALM score
Source: Ann Intensive Care. 2018 Apr 18;8:46. doi: 10.1186/s13613-018-0395-5 (PMC5906418; doi:10.1186/s13613-018-0395-5)
Supplement: Supplementary file 1 — Additional file 1: Table S1. Type of cardiac surgery. [file 13613_2018_395_MOESM1_ESM.docx]

| Type of cardiac surgery, n (%) | Ischaemic group  (n = 48) | Non ischaemic  (n = 96) | *p* value |
| --- | --- | --- | --- |
| Aortic valve replacement | 19 (39.6) | 41 (42.7) | 0.72 |
| Mitral valve replacement | 10 (20.8) | 20 (20.8) | 0.99 |
| Coronary artery bypass ≤ 2 | 23 (47.9) | 44 (45.8) | 0.82 |
| Coronary artery bypass >2 | 5 (10.4) | 14 (14.6) | 0.49 |
| Tricuspid valve repair, n (%) | 1 (2.1) | 4 (4.2) | 0.52 |
| Mitral valve repair, n (%) | 4 (8.3) | 6 (6.3) | 0.64 |

**Additional file 1: Table S1: Type of cardiac surgery.**
