# Supplementary material for: Biological scoring system for early prediction of acute bowel ischemia after cardiac surgery: the PALM score
Source: Ann Intensive Care. 2018 Apr 18;8:46. doi: 10.1186/s13613-018-0395-5 (PMC5906418; doi:10.1186/s13613-018-0395-5)
Supplement: Supplementary file 2 — Additional file 2: Table S2. Postoperative course. [file 13613_2018_395_MOESM2_ESM.docx]

**Additional file 2: Table S2: Postoperative course.**

| **Outcome** | **Ischemia group**  **N = 48** | **Non ischemia group**  **N = 96** | ***p* value** |
| --- | --- | --- | --- |
| **SAPS II score, mean ± SD** | 49.6 ± 13.1 | 36.1 ± 11.3 | <0.001 |
| **Pneumonia, n (%)** | 17 (35.4) | 20 (20.8) | 0.06 |
| **Acute kidney injury, n (%)** | 43 (89.6) | 48 (50.0) | <0.001 |
| **Renal replacement therapy, n (%)** | 24 (50.0) | 6 (6.3) | <0.001 |
| **Mortality, n (%)** | 39 (81.3) | 7 (7.3) | <0.001 |
