# Supplementary material for: Biological scoring system for early prediction of acute bowel ischemia after cardiac surgery: the PALM score
Source: Ann Intensive Care. 2018 Apr 18;8:46. doi: 10.1186/s13613-018-0395-5 (PMC5906418; doi:10.1186/s13613-018-0395-5)
Supplement: Supplementary file 3 — Additional file 3: Table S3. Features for the distinction between mesenteric ischemia and ischemic colitis. [file 13613_2018_395_MOESM3_ESM.docx]

| **Variables** | **Mesenteric ischemia**  **(n = 31)** | **Ischemic colitis**  **(n = 17)** | ***p*  value** |
| --- | --- | --- | --- |
| ***Clinical features*** |  |  |  |
| Shock, n (%) | 15 (48.4) | 5 (29.4) | 0.20 |
| Abdominal pain, n (%) | 14 (45.2) | 6 (35.3) | 0.51 |
| Rectal bleeding, n (%) | 1 (3.2) | 5 (29.4) | **0.02** |
| Diarrhea, n (%) | 2 (6.5) | 2 (11.8) | 0.61 |
| Bowel obstruction, n (%) | 5 (16.1) | 2 (11.8) | 0.69 |
| Other, n (%)* | 4 (12.9) | 1 (5.9) | 0.64 |
| ***Biological features*** |  |  |  |
| Liver cytolysis, n (%) | 1 (3.2) | 1 (5.9) | 0.82 |
| Metabolic acidosis, n (%) | 2 (6.5) | 5 (29.4) | 0.08 |
| Hyperlactatemia, n (%) | 5 (16.1) | 3 (17.6) | 0.89 |
| ***Paraclinical features*** |  |  |  |
| ***Colonoscopy*, n (%)***  No mucosa damage  Stage I  Stage II  Stage III | 11 (35.5)  3 (27.3)  0 (0)  2 (18.2)  6 (54.5) | 13 (76.5)  0 (0)  2 (15.4)  1 (7.7)  10 (76.9) | **0.006**  0.08  0.48  0.57  0.39 |
| ***CT scan, n (%)***  Normal results | 18 (58.1)  8 (44.4) | 7 (41.2)  4 (57.1) | 0.26  0.67 |
| ***Surgical exploration, n (%)*** | 29 (93.5) | 11 (64.7) | **0.02** |
| Normal  Necrosis  Ischemia  Perforation | 2 (6.9)  14 (48.3)  17 (58.6)  3 (10.3) | 0 (0)  4 (36.4)  9 (81.9)  1 (9.0) | 0.87  0.72  0.48  0.93 |
| ***Surgery only based on clinical and biological features, n (%)*** | 6 (19.4) | 3 (17.6) | 0.82 |

**Additional file 3: Table S3: Features for the distinction between mesenteric ischemia and ischemic colitis.**
